# Visuospatial Neglect - a Theory-Informed Overview of Current and Emerging Strategies and a Systematic Review on the Therapeutic Use of Non-invasive Brain Stimulation

**DOI:** 10.1007/s11065-019-09417-4

**Published:** 2019-11-20

**Authors:** Paul Theo Zebhauser, Marine Vernet, Evelyn Unterburger, Anna-Katharine Brem

**Affiliations:** 1 Department of Neuropsychology, Max-Planck Institute of Psychiatry, Kraepelinstrasse 2-10, 80804 Munich, Germany; 2 Department of Psychiatry and Psychotherapy, Klinikum Rechts der Isar der Technischen Universität, Munich, Germany; 3 Section on Neurocircuitry, Laboratory of Brain and Cognition, NIMH/NIH, Bethesda, MD USA; 4 Division of Neuropsychology, Universitätsklinik Zürich USZ, Frauenklinikstrasse 26, Zurich, Switzerland; 5 Berenson-Allen Center for Noninvasive Brain Stimulation, Department of Neurology, Beth Israel Deaconess Medical Center, Harvard Medical School, Boston, MA USA

**Keywords:** Visuospatial neglect, Diagnosis, Treatment, Noninvasive brain stimulation, Transcranial magnetic stimulation, Transcranial electric stimulation, Cognitive rehabilitation

## Abstract

Visuospatial neglect constitutes a supramodal cognitive deficit characterized by reduction or loss of spatial awareness for the contralesional space. It occurs in over 40% of right- and 20% of left-brain-lesioned stroke patients with lesions located mostly in parietal, frontal and subcortical brain areas. Visuospatial neglect is a multifaceted syndrome - symptoms can be divided into sensory, motor and representational neglect - and therefore requires an individually adapted diagnostic and therapeutic approach. Several models try to explain the origins of visuospatial neglect, of which the “interhemispheric rivalry model” is strongly supported by animal and human research. This model proposes that allocation of spatial attention is balanced by transcallosal inhibition and both hemispheres compete to direct attention to the contralateral hemi-space. Accordingly, a brain lesion causes an interhemispheric imbalance, which may be re-installed by activation of lesioned, or deactivation of unlesioned (over-activated) brain areas through noninvasive brain stimulation. Research in larger patient samples is needed to confirm whether noninvasive brain stimulation can improve long-term outcomes and whether these also affect activities of daily living and discharge destination.

## Introduction

Stroke is one of the leading reasons for long-term disabilities in adults and is responsible for large and increasing health-costs in the United States, projecting to more than double from $36.7 billion in 2015 to $94.3 billion in 2035 (Benjamin et al., 2018). Visuospatial neglect is a frequent and disabling condition after stroke and is characterized by reduction or loss of spatial awareness for the contralesional space. Visuospatial neglect adversely affects rehabilitation outcome of other stroke symptoms (Buxbaum et al., 2004) and significantly impairs Activities of Daily Living (ADL: Di Monaco et al., 2011). The overall recovery pattern is slower and more attenuated (Katz, Hartman-Maeir, Ring, & Soroker, 1999), and the length of stay in inpatient rehabilitation is longer than in stroke patients without visuospatial neglect (Gillen, Tennen, & McKee, 2005). Moreover, visuospatial neglect may have an impact on discharge destination, that is, home versus care facility (Chen, Hreha, Kong, & Barrett, 2015; Wee & Hopman, 2005). Spontaneous recovery from visuospatial neglect-symptoms occurs in the first months after stroke, however, in approximately 40% of patients symptoms persist (Nijboer, Kollen, & Kwakkel, 2013). This symptom persistence emphasizes the need for further investigation of existing therapeutic means and the development of novel treatment methods.

Though visuospatial neglect-symptoms are more prevalent after right-hemispheric stroke, ranging from 33% to 82%, occurrence is nevertheless high after left-hemispheric stroke, ranging from 20% to 67% (Bowen, McKenna, & Tallis, 1999), justifying specific consideration in diagnostics and rehabilitation programs for patients with left brain damage stroke also (Beis et al., 2004). Considering that in general, left-hemispheric stroke is more easily recognized (Portegies et al., 2015), presumably due to more evident symptoms like aphasia, the overall presence of visuospatial neglect-symptoms is likely underestimated. Notably, reported prevalence rates also depend on the sensitivity of the diagnostic tools used (Bowen et al., 1999). Nevertheless, the higher prevalence as well as the possibly more severe and persistent character of visuospatial neglect after right-side brain damage (Ringman, Saver, Woolson, Clarke, & Adams, 2004) is explained by several theories of visual attention.

The present review will provide an up-to-date overview of models, anatomy, diagnosis and treatment of visuospatial neglect. Finally, the use and potential of noninvasive brain stimulation as a novel treatment protocol will be reviewed systematically and discussed comprehensively.

## Neglect Definition and Symptomatology

Neglect can be divided into sensory, (pre-)motor, and representational (imaginal) neglect. It can furthermore affect different ranges of space (personal, peripersonal, extrapersonal space) and frames of references (egocentric, allocentric). Some authors combine said classifications and describe for example, extrapersonal neglect in terms of visual or auditory (i.e., sensory) deficits and personal neglect in terms of somatosensory deficits (Thiebaut de Schotten et al., 2014). In clinical practice, subtypes usually overlap, and patients present with mixed symptoms.

Visuospatial neglect is the most important neglect subtype and is subsumed under sensory neglect. It is a higher-order, multifaceted, supramodal cognitive deficit, which affects space-related behaviour not caused by an elementary sensorimotor deficit. Resulting clinical characteristics and symptoms are described below.

### Sensory Neglect

Sensory neglect is described as a selective unawareness of sensory (visual, auditory, tactile, and olfactory) stimuli (Rode, Pagliari, Huchon, Rossetti, & Pisella, 2017). Patients show unilaterally reduced attention and spontaneous exploration and fail to respond to stimuli presented in the contra-lesional hemi-space (see below for clinical signs and symptoms). Visuospatial neglect symptoms are the most obvious and the most important with regard to their impact on neurorehabilitation and ADL.

### Motor or Premotor Neglect

Motor neglect is characterized by reduced spontaneous use or a complete nonuse of contralesional limbs towards or within the contralesional hemi-space in the absence of hemiplegia, increased muscle tone, or pyramidal signs (Sampanis & Riddoch, 2013). Motor neglect can occur independently of visuospatial neglect (Punt, Riddoch, & Humphreys, 2005) and includes symptoms of pseudohemiparesis or limb akinesia (hemiparetic symptomatology despite an intact corticospinal tract), directional akinesia, or hypokinesia (reduced head, arm, or eye movements toward contralesional space), hypometria (reduced amplitude in movement in the affected space, e.g., directional hypometria of the eyes shows itself in multiple small saccades before reaching a target), or motor impersistence (inability to sustain a movement or posture in the affected space). Though all of these symptoms are summarized under the term “motor neglect” according to their appearance, they are tightly associated with attention functions, that is, patients are able to perform regular movements when asked to do so (Garbarini, Piedimonte, Dotta, Pia, & Berti, 2013). Notably, motor neglect can hamper rehabilitation of motor functions, as patients often do not sufficiently exercise their paretic limbs.

### Representational (Imaginal) Neglect

Neglect can manifest itself in the absence of external stimuli, that is, when imagining visual scenes. For example, Bisiach and Luzatti (1978) asked patients to describe the “Piazza del Duomo” in Milan (the patients’ home town). When patients imagined standing on one side of the Piazza, they described the features situated on the right, when they imagined standing on the opposite side, they did the same and now mentioned the features they had previously omitted. They could recall the details from their memories but were only able to access the features of one side of the representation of the Piazza depending on their imagined location. While representational neglect in combination with visuospatial neglect is not uncommon (Cecilia Guariglia, Palermo, Piccardi, Iaria, & Incoccia, 2013), it may also occur solely in imagined space. This is contradictory to the view that visual imagery and spatial perception share the same neural substrates. The lesions of patients with “pure” representational neglect are inconsistent and include the right frontal lobe (Guariglia, Padovani, Pantano, & Pizzamiglio, 1993), the right thalamus (Ortigue et al., 2001), and the right parietal lobe (Beschin, Cocchini, Della Sala, & Logie, 1997), suggesting the involvement of widespread networks and white matter tracts (Boccia et al., 2018). However, “pure” representational neglect is rather rare, maybe because it is not tested for, or because imagined space contains less attention-capturing elements (Bartolomeo, Bachoud-Lévi, Azouvi, & Chokron, 2005).

As there is evidence for number-space associations in humans (Dehaene, Bossini, & Giraux, 1993), biases regarding the spatial representation of numbers along a mental number line may be expected in visuospatial neglect. For instance, deficits were found in responding to smaller numbers (Patrik Vuilleumier, Ortigue, & Brugger, 2004) and in the bisection of aurally presented number lines in patients with visuospatial neglect (Zorzi, Priftis, Meneghello, Marenzi, & Umiltà, 2006; Zorzi et al., 2012). However, the latter finding is the matter of a controversial debate, as double dissociations between a bias in the bisection of number intervals and physical lines were observed (van Dijck, Gevers, Lafosse, Doricchi, & Fias, 2011). It is still not unequivocally clear how number lines are mentally represented, in both healthy adults and patients with visuospatial neglect (Mihulowicz, Klein, Nuerk, Willmes, & Karnath, 2015).

### Range of Space

Neglect can be divided into personal (body space), peripersonal (reaching space), and extrapersonal (far space) neglect. Those different radii around the subject can be effected selectively, though this is rather rare. A recent study found prevalence rates for single-domain neglect ranging from 11% (only extrapersonal) to 22% (only peripersonal: Spaccavento, Cellamare, Falcone, Loverre, & Nardulli, 2017). Noteably, the term “extrapersonal” is sometimes applied to visual and auditory (and theoretically olfactory) sensations, whereas the term “personal” would only refer to somatosensory and therefore haptic or proprioceptive information (Thiebaut de Schotten et al., 2014).

*Personal neglect* refers to a lack of exploration or use of contralesional limbs or the head. Patients can appear as being hemiparetic. Their body posture and gaze direction are often deviated towards the ipsilesional hemi-space. Reactions to touch or painful stimuli can be impaired. For instance, if the hand of a patient gets caught in the wheels of the wheelchair the patient may not show any overt reaction. Patients may only shave the left half of their face, clean the right part of their body, or put make-up on the left half of their face.

*Peripersonal neglect* can cause patients to eat food only from the left half of the plate. Only when the plate is turned, patients are able to continue their meal and may make remarks such as - “Did you put more food on my plate?” Similar reactions can be observed when patients perform cancellation tests. Visual exploration during cancellation tasks commonly occurs from right to left and can be accompanied by exclamations of surprise when a new target is perceived (e.g., “Did you just put that letter there?” or “Did you trick me?”). Similarly, patients often omit or misread words or letters on the contralesional side of pages or words (often referred to as “neglect dyslexia”).

Patients with *extrapersonal neglect* often overlook people and objects in the left hemi-space. They may bump into obstacles or doorframes. Serious difficulties can occur when they move outdoors, for example, crossing a street can become dangerous, as oncoming cars are not perceived on time. Patients therefore may not be able to move independently at all outside their home.

### Frame of Reference

Cortical representations of space are not limited to viewer-centered reference frames, for which reason visuospatial neglect symptoms typically occur with respect to spatial coding systems that are more complex than a simple “Cartesian” coordinate system dividing space along two or three axes around the viewer. In visuospatial neglect, at least two different coding systems can be impaired.

In *egocentric or viewer-centered neglect*, coding in relation to egocentric coordinates, with the own body as the center of the reference frame, is impaired. This can be described as an inability to orient to stimuli that are situated in the contralesional hemi-space with respect to the midsagittal plane of the body.

In *allocentric neglect*, patients fail to allocate attention to the contralesional half of an object, regardless of object location. This deficit can be revealed when copying figures.

Evidence to date disagrees on whether egocentric and allocentric neglect are dissociated (Hillis et al., 2005) or correlated (Rorden et al., 2012), which can probably best be accounted for by the use of different diagnostic batteries.

## Related Symptoms and Syndromes

Visuospatial neglect is often associated with large lesions and commonly occurs concomitantly with other deficits **(**homonymous hemianopia, motor deficits, or extinction phenomena), which complicates diagnosis. Moreover, symptoms underlying other deficits can lead to an impression of visuospatial neglect and severity of single subsymptoms can vary greatly requiring careful diagnostic procedures.

### Homonymous Hemianopia

The term homonymous hemianopia refers to visual field loss on one side of the vertical midline due to unilateral post-chiasmatic brain lesions. Visuospatial neglect and homonymous hemianopia co-occur often and symptoms can appear similar. In homonymous hemianopia, patients typically suffer from hemianopic alexia and problems in visual exploration, while spatial representations of both hemi-spaces are intact. Differentiation can be based on affected modalities (only visual modality is affected in homonymous hemianopia), visual behaviour, covert attention, and performance in cancellation and drawing tests (see Table 1). Note, that in homonymous hemianopia the defect strictly follows the midline, whereas visuospatial neglect is characterized by a gradual fading towards the contralesional space (Behrmann, Watt, Black, & Barton, 1997; Butler, Eskes, & Vandorpe, 2004).

**Table 1:** Differences between visuospatial neglect symptoms and homonymous hemianopia

	Visuospatial neglect	Homonymous hemianopia
Lesions	Fronto-parietal lesions (mainly territory of middle cerebral artery).	Postchiasmatic lesions of the visual tract or occipital lesions (mainly territory of posterior cerebral artery).
Awareness for deficits	Awareness for deficits is reduced (anosognosia), contralesional parts of the body, the external and internal world seem not to exist anymore. Anosodiaphoria can occur.	Awareness for deficits can be reduced initially but improves quickly. Anosognosia and anosodiaphoria are rare.
Modalities	Multimodal deficits can occur (visual, auditory, tactile, motor, olfactory).	Restricted to deficits of the visual modality.
Visual behaviour	Lack of attention to contralesional hemispace, independent of gaze direction. Deviation of gaze, head and sometime upper body towards the ipsilesional side. Reduced eye contact with conversational partner.	Loss of contralesional visual field with respect to the position of the head and eyes. Compensational eye and head movements towards the contralesional hemispace.
Drawing and cancellation	Contralesional omissions in drawing or cancellation tests.	Drawing and cancellation tests are mostly unaffected.
Line bisection	Ipsilesional deviation.	Contralesional deviation occurs frequently.
Attention (Posner paradigm)	Attentional shift is impaired.	Attentional shift is not impaired.
Compensation	Cueing on the contralesional side can lead to a transient improvement.	Compensation of visual deficits with head- and eye-movements occurs spontaneously (over-compensation may occur). Cueing does not have an impact.
Central fixation	Difficulty in maintaining central fixation.	Central fixation is not impaired.
VEPs	Near normal response of VEPs. Prolonged latency can occur on affected side.	Different response of VEPs in ipsi- and contralesional visual field.
Extinction	Visual extinction is commonly associated.	Visual extinction is not commonly associated.

Adapted from (Kerkhoff & Schindler, 1997) and (Ting et al., 2011)

*VEP* Visually evoked potential

### Extinction

Extinction is a relatively frequent consequence of unilateral brain damage and occurs in 24% of patients with right hemispheric lesions (Becker & Karnath, 2007), with a slightly higher prevalence following middle compared to posterior cerebal artery stroke (Chechlacz, Rotshtein, Demeyere, Bickerton, & Humphreys, 2014). It can be defined as “an inability to process or attend to the more contralesionally located stimulus when *two* stimuli are simultaneously presented (visual extinction), or when *two* actions have to be performed with both hands simultaneously (motor extinction)” (Kerkhoff, 2001). In clinical routine, visual extinction can easily be detected by first confronting the patient using a single unilateral stimulus (e.g., a moving finger on the right side) and afterwards with bilateral double stimulation. A patient suffering from extinction is able to detect a single stimulus on the contralesional side, however the same stimulus would become “extinguished” in case of double stimulation. Extinction can occur in a single modality but can also have crossmodal effects (e.g., concomitant tactile and visual stimuli: Mattingley, Driver, Beschin, & Robertson, 1997).

Extinction and visuospatial neglect frequently co-occur after large lesions due to middle cerebral artery-infarction. Nevertheless, they can be differentiated rather well, as visuospatial neglect can already be observed when only one stimulus is present. Furthermore, typical clinical features of visuospatial neglect such as ipsilesional head and gaze orientation are absent in extinction (de Haan, Karnath, & Driver, 2012).

### Hemiparesis

The unilateral loss or weakening of motor functions present in hemiparesis following stroke can sometimes resemble severe forms of motor neglect. The measurement of motor evoked potentials informs about impairments of the corticospinal pathway and allows differentiating hemiparesis and motor neglect. However, these impairments often co-occur due to extensive lesions.

## Modulation of Visuospatial Neglect Symptoms by Other Cognitive Functions

Widespread lesions causing visuospatial neglect frequently entail co-occurrence of other cognitive dysfunctions that inevitably influence visuospatial neglect in a reciprocal fashion. In order to appropriately diagnose and treat symptoms of visuospatial neglect it is therefore crucial to assess other cognitive functions and elucidate their contribution to clinical symptoms and rehabilitation.

### The Role of Non-spatial Attention in Visuospatial Neglect

Patiens with visuospatial neglect often exhibit additional non-spatial deficits in attention, which can exacerbate spatial symptoms and should therefore be taken into account as a substantial feature of visuospatial neglect.

For instance, reduced galvanic skin responses to electrical stimulation (K. M. Heilman, Schwartz, & Watson, 1978) and significantly increased reaction times to ipsilesional auditory stimuli (Samuelsson, Elisabeth Hjelmquist, Jensen, Ekholm, & Blomstrand, 1998) suggest a distinct non-spatial impairment of arousal and alertness in patients with visuospatial neglect. Arousal and alertness could moreover be increased by exerting time pressure, which improved visuospatial neglect symptoms (George, Mercer, Walker, & Manly, 2008). The role of arousal or alertness in visuospatial neglect is furthermore supported by the finding that visuospatial neglect symptoms re-occur after administration of the sedative midazolam (Lazar et al., 2002). Similar associations and modulatory influences have been reported for sustained attention (Malhotra, Coulthard, & Husain, 2009) and selective attention (Husain, Shapiro, Martin, & Kennard, 1997).

Anatomical evidence for the strong association between visuospatial neglect and non-spatial attention deficits is provided by a number of studies showing bidirectional effects of spatial-attention networks and the ascending reticular activating system (for a review, see Boukrina & Barrett, 2017).

### The Role of Working Memory and Executive Functions in Visuospatial Neglect

Executive dysfunctions can be observed in typically administered cancellation tasks, in which targets must be crossed out within an array of distractors. In addition to missing targets on the contralesional side, patients sometimes also fail to cancel targets on the ipsilesional side (Danckert & Ferber, 2006), which can be interpreted as an inefficient search strategy. More suggestive of an executive deficit is the so-called “revisiting” behavior, where patients repeatedly cancel out the same target (Husain & Rorden, 2003). Repeated allocation of attention to the same stimulus may be related to similar processes as can be observed in patients with severe executive dysfunctions, who show perseverative tendencies, “magnetic” behaviour related to object-inherent functionality (e.g., repeatedly pressing a light-switch), and impaired inhibition (desinhibition and involuntary repetition of external and internal actions and thoughts). Such behaviour may be related to dysfunctions of fronto-striatal, or fronto-parietal pathways, respectively. In the context of a primarily visuospatial neglect-related impairment these symptoms may be regarded as a space-related executive dysfunction or an impairment in spatial working-memory (Striemer, Ferber, & Danckert, 2013).

### Awareness of Deficits

Anosognosia can be defined as a lack of awareness of deficits or illness (McGlynn & Schacter, 1989). Anosognosia poses a substantial problem in rehabilitation efforts (Adair et al., 1995), and is a predictor of deficits in ADL after stroke (Vossel, Weiss, Eschenbeck, & Fink, 2013). Being present in about 10% of acute stroke patients (Starkstein, Jorge, & Robinson, 2010), it is mainly associated with unilateral right-sided lesions, specifically lesions in deep white matter structures and the basal ganglia, which are also involved in executive functions. In some cases, anosognosia is accompanied by confabulations, for example, if patients are asked to explain how they could miss all the left-sided targets on a cancellation task they would come up with various reasons such as “I was tired”, or “This task was boring” and so on. As confabulations often occur in patients with executive dysfunctions, this behaviour may be explained by concomitant executive dysfunctions. Unawareness may also be regarded as an extreme dysfunction of Theory of Mind for the Self (Besharati et al., 2016). Explanatory theories of anosognosia after stroke include psychodynamic approaches, state-dependent learning, the presence of a confusional state, failure of sensory feedback, and others (for a review, see Heilman, 2014). Awareness of deficits usually improves with time, however, patients might continue to trivialize the extent and the sequelae of impairments, a state referred to as “anosodiaphoria” (Critchley, 1957).

## Theoretical and Functional-Anatomical Models

Theoretical models vary regarding their proposed physiological mechanisms, however, most models focus on the differences in the prevalence of right- and left-sided visuospatial neglect. Though it is difficult to distinguish between these models on a behavioural level, some of them are mutually compatible (Sack, 2010).

As these models mainly concentrate on specific visual aspects of visuospatial neglect, they lack a comprehensive integration of the multitude of underlying symptoms. However, technological advances in neuroimaging and brain stimulation recently stimulated this field of research, allowing development of more sophisticated network-driven models based on functional anatomy (Corbetta & Shulman, 2011).

### Attention-Shifting and Disengagement of Attention

Various models of attentional control and attention-shifiting such as the “moving-spotlight” hypothesis (e.g. Crick, 1984) or the “activity-distribution” model (LaBerge, Carlson, Williams, & Bunney, 1997) offer more or less tangible explanations for the phenomenon of visuospatial neglect. Arguably, the most influential theory within this context is the attention-shifting model proposed by Posner, Walker, Friedrich, and Rafal (1984), which postulates that covert attention shifts (i.e., without muscular involvement) depend on three internal mental operations: (1) disengagement of attention from a current stimulus, (2) shift of attention to the new target, and (3) engagement of the new target. According to this model, in visuospatial neglect, damage to the parietal lobe results mainly in a deficit of disengagement, which is expressed as an increased reaction latency or even failure to respond to contralesional targets. Notably, patients can nonetheless perform movements in all directions without any direction-specific disturbances, if movements are performed “top-down” (Karnath, 2015).

### Biased Body-Centered Matrix

Karnath (2015) emphasized the role of disturbed input transformation in the aetiology of visuospatial neglect, which leads to a deviation of egocentric space representation towards the ipsilesional side. This explanation is based on the assumption that in order to obtain a stable representation of the sorroundings, an individual integrates information from different peripheral sources, and performs spatially oriented actions in relation to a specific body reference. This model explains the ipsilesional gaze-deviation and head-to-trunk-orientation frequently observed in patients with visuospatial neglect.

### Heilman’s and Mesulam’s Hemispatial Theory

It has been suggested that the unequal distribution of left- versus right-sided visuospatial neglect indicates a hemispheric asymmetry in attention. Heilman and van den Abell (1980) observed desynchronisation in EEG of the left parietal lobe after presentation of right-lateralized stimuli, while the right parietal lobe desynchronised equally after right- or left-lateralized stimuli, suggesting right-hemispheric dominance in spatial attention. Based on these findings, Mesulam (1981, 1999) proposed that visuospatial neglect is more frequent after right brain damage because the left hemisphere controls attention only towards the right hemi-space, while the right hemisphere controls attentional orienting toward both the left and right hemi-spaces.

### Interhemispheric Rivalry Model

Kinsbourne (1970, 1987, 1993) proposed that allocation of spatial attention is balanced by mutual transcallosal inhibition resulting in a competition of the hemispheres to direct attention to the contralateral side. A lesion-induced imbalance within this competitive attentional network accordingly leads to hypoactivity of the lesioned hemisphere and hyperactivity of the intact hemisphere. The predominance of visuospatial neglect after right-hemisphere lesions is in accordance with the “attention hypothesis” (Kinsbourne, 1970; Posner et al., 1984), which postulates an asymmetrical distribution of brain activity when orienting attention. Clinical observations provided intriguing evidence in support of this model. Vuilleumier and colleagues observed visuospatial neglect symptoms in a patient after a (first) right-hemispheric stroke (affecting the angular gyrus), which then remitted after a (second) left-hemispheric stroke (affecting the frontal eye field: P Vuilleumier, Hester, Assal, & Regli, 1996). Subsequently, several studies in animals and humans examined this principle of two competing hemispheric “processors” of spatial attention by means of noninvasive brain stimulation. Converging evidence points to the potential of noninvasive brain stimulation to restore interhemispheric balance and ameliorate visuospatial neglect symptoms. Either by enhancing the activity of remaining neurons or by reducing the excitability of, and thus the inhibitory competition by, intact structures in the contralateral hemisphere.

### Functional-Anatomical Models

Visuospatial neglect is a heterogeneous syndrome, suggesting complex anatomical backgrounds. In rare cases, visuospatial neglect can be caused by trauma (McKenna, Cooke, Fleming, Jefferson, & Ogden, 2006), neurodegenerative disease (Andrade et al., 2010), or neoplasia (Yoon & Pirris, 2013), but in the vast majority of patients it is caused by middle - or less frequently - posterior cerebral artery stroke. Visuospatial neglect after stroke, like motor and language deficits (Corbetta et al., 2015), not only arises from cortical lesions, but via disconnection of widespread networks, leading to diaschisis and resulting in cortical hypoperfusion and changes in interhemispheric balance.

Regarding cortical lesions, visuospatial neglect is traditionally interpreted as a parietal sign (Vallar & Perani, 1986). However, the importance of frontal (M. Husain & Kennard, 1996) and subcortical gray matter nuclei (Karnath, Himmelbach, & Rorden, 2002) damage has been acknowledged. Clinical evidence as well as results from neuroimaging studies point to various critical cortical regions like the temporo-parietal junction (Leibovitch et al., 1998; Vallar & Perani, 1986), the angular (Hillis et al., 2005) and supramarginal gyri (Doricchi & Tomaiuolo, 2003) and the posterior parietal (Azouvi et al., 2002), the superior temporal (H. O. Karnath, Ferber, & Himmelbach, 2001) and the ventrolateral frontal cortex (Rengachary, He, Shulman, & Corbetta, 2011). These studies showed partly inconsistent and even conflicting results, reflecting methodological and patient-sampling differences. Furthermore, the heterogenity of described cortical lesions raises questions about the involvement of underlying non-cortical structures in visuospatial neglect.

Recent functional imaging studies in healthy volunteers and visuospatial neglect-patients suggest that spatial cognition and attention are subserved by different spatio-temporal interactions within a widespread fronto-parietal network along the above-mentioned temporo-parietal and frontal cortical nodes (Doricchi, Thiebaut de Schotten, Tomaiuolo, & Bartolomeo, 2008; Sack, 2010; Thiebaut de Schotten et al., 2014). Corbetta and Shulman (2011) developed an elaborate functional-anatomical network of visuospatial neglect around separate but interacting sub-networks of visuospatial attention. They propose (a) a bilateral dorsal fronto-parietal network along the frontal eye field and the posterior parietal cortex (PPC), which executes shifts of spatial attention and (b) a right-lateralized ventral fronto-parietal network involving the temporo-parietal junction and the ventral frontal cortex, which mediates reactions to unexpected events or stimuli. In this framework, lesions to the ventral system are seen as the leading cause for visuospatial neglect.

Functional-anatomical network models stress the importance of white-matter disconnections and provide new momentum for the pioneering work of Geschwind, who interpreted visuospatial neglect as a disconnection-syndrome (Geschwind, 1965a, b). Important *intra*hemispheric white matter tracts in visuospatial neglect include the superior longitudinal fasciculus, the arcuate fasciculus, and the inferior and superior occipitofrontal fasciculi (Karnath, Rorden, & Ticini, 2009; Thiebaut de Schotten et al., 2014). *Inter*hemispheric disconnection can lead to visuospatial neglect as well. Animals (Gaffan & Hornak, 1997) and stroke patients (Tomaiuolo et al., 2010) have shown severe signs of visuospatial neglect after selective damage to the splenium of the corpus callosum.

Additionally, it has to be pointed out that a purely disconnective account of visuospatial neglect may not be sufficient (Doricchi et al., 2008). Hillis et al. (2002) found that, independent of subcortical lesion localization, visuospatial neglect was only present if there was an associated *cortical* hypoperfusion. They conclude that a subcortical disruption of fronto-parietal connections may cause visuospatial neglect by reducing functional activity in the entire cortico-subcortical fronto-parietal network.

Several authors sought to link certain lesion patterns to subtypes or symptoms of visuospatial neglect. Verdon, Schwartz, Lovblad, Hauert, and Vuilleumier (2010) found specific neural correlates related to perceptive (right inferior parietal lobule), exploratory (right dorsolateral prefrontal cortex), and object-centred (deep temporal lobe) aspects of visuospatial neglect. Overlap analyses indicated that subcortical lesions of paraventricular white matter tracts were associated with more severe visuospatial neglect. Doricchi and Tomaiuolo (2003) proposed that different visuospatial neglect signs depend on selective lesions of white matter fibres and resulting dysfunction of corresponding different cortical modules. Mesulam (2002) suggested that lesions within the fronto-parietal-cingulate network are likely to cause multimodal visuospatial neglect, whereas lesions resulting in a specific disconnection from sensory or motor areas could lead to a variety of modality-specific visuospatial neglect symptoms. In recent years, meta-analytic approaches using activation-likelihood-estimations shed further light on the link between lesion patterns and subtpyes of visuospatial neglect (Chechlacz, Rotshtein, & Humphreys, 2012) and performance on visuospatial neglect tests (Molenberghs, Sale, & Mattingley, 2012), but the neuroanatomical basis of the heterogenitiy of visuospatial neglect remains a matter of uncertainty.

To summarize, visuospatial neglect symptoms not only arise from actual lesions but via intra-hemispheric disconnection affecting fronto-parietal networks and leading to diaschisis effects giving way to hypoperfusion of relevant cortical areas as well as subsequent changes in interhemispheric balance. Despite the converging evidence about which brain areas and functional networks contribute to visuospatial neglect symptoms, the underlying mechanisms are still unclear. Novel neuroimaging and stimulation techniques may shed more light on these in the future. It is to note, that the superior temporal resolution of electroencephalography (EEG) seems to be a promising tool to further elucidate functional relations of anatomically relevant regions in visuospatial neglect (Fellrath, Mottaz, Schnider, Guggisberg, & Ptak, 2016; Yordanova et al., 2017).

## Diagnosis

Due to large symptom variability and heterogenous presentations, neuropsychological diagnosis should be based on patient observation and a comprehensive test battery covering a broad range of symptoms, then guiding subsequent neurorehabilitative efforts. In the US (Winstein et al., 2016), the UK (Intercollegiate Stroke Working Party, 2012), and Germany (H.-O. Karnath & Zihl, 2012) guidelines recommend a multidisciplinary approach concerning the assessment of visuospatial neglect.

Despite its high prevalence, visuospatial neglect is often overlooked at hospital admission for several reasons. Firstly, most patients suffer from additional cognitive and motor impairments that are more acute and more easily recognized. Secondly, visuospatial neglect affects different modalities and severity can vary greatly. Thirdly, brief screening procedures, often performed due to lack of time (e.g., Mini-Mental-State-Examination), lack appropriate measures to detect visuospatial neglect reliably, and finally, medical staff are often not appropriately trained to assess visuospatial neglect. Systematic screening is crucial for planning rehabilitative efforts and can significantly improve rehabilitation outcome in visuospatial neglect patients (Edwards et al., 2006).

**Fig. 1:**
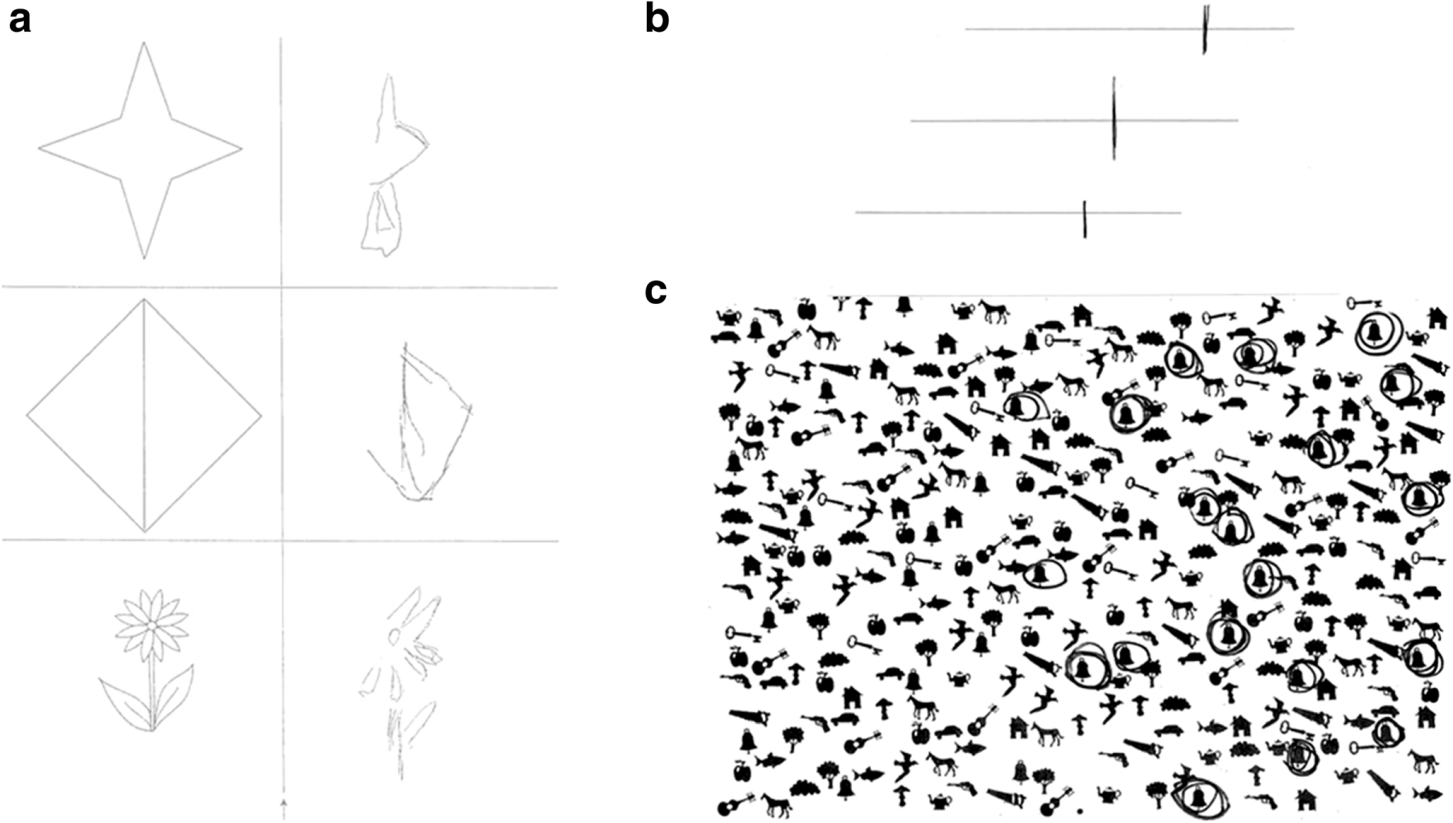
Typically administered paper-pencil-tests in visuospatial neglect. **a** Copying of simple figures: Contralesional omissions of details or object parts are often seen in visuospatial neglect; **b** Line bisection: The patient is asked to set a mark through the midpoint of a horizontal line; ipsilesional bias indicates visuospatial neglect; **c** Bells Test: In cancellation tasks, patients are asked to find and mark all targets (in this case, bells) in an array of distractors. Patients with severe visuospatial neglect find targets only on the ipsilesional side

Since normal performance in a single test, such as a reading or line bisection, does not exclude the presence of visuospatial neglect, it is vital that different tests covering a number of symptoms are administered. For example, a visuospatial neglect test battery, such as the Behavioural Inattention Test (BIT, Wilson et al., 1987), integrates a number of tests that should be complemented with assessments of ADL. Other methods such as oculography and eye tracking, virtual reality and other computerized attention tests can further elucidate specific aspects of visuospatial neglect (Fig. 1, Table 2). In many patients, performance on neglect tests is highly inconsistent over time (Hamilton, Coslett, Buxbaum, Whyte, & Ferraro, 2008), probably reflecting fluctuations in basic attentional functions like arousal and alertness.

**Table 2:** Diagnostic instruments used for visuospatial neglect symptoms

Tests	Brief description	Comments and recommendations
Cancellation	Crossing out visual targets on a paper sheet. Omissions of contralesional targets indicates visuospatial neglect.	Good sensitivity (Marsh & Kersel, 1993). Qualitative interpretation enhances the detection rate. Starting point is most sensitive to visuospatial neglect.
Line bisection	Marking the middle point of horizontal lines. Ipsilesional deviation indicates visuospatial neglect.	Easy and fast administration, used for screening rather than for clinical diagnosis. Good retest reliability (Facchin, Beschin, Pisano, & Reverberi, 2016). Note, that false positive results can result from HH.
Copying figures and drawing	Copying (e.g., flower, house etc.) or drawing objects (e.g., clock). Omissions or cramming of contralesional details indicates visuospatial neglect.	Assesses mainly representational aspects of visuospatial neglect. Interpretation is limited by subjectivity.
Reading	Reading a paragraph of text. Omissions of words or letters at the contralesional side of a line or a word indicate visuospatial neglect.	Assesses ‘Neglect dyslexia’.
BIT (Behavioural inattention test)	Compilation of 17 subtests (such as the ones described above, and tasks related to ADL-functions). An overall index can be calculated.	Assesses functional performance.
Catherine Bergego scale	10-item checklist that assesses visuospatial neglect-specific behavior in ADL.	Widely used in clinical studies.
Comb and Razor test	The patient is asked to comb their hair and shave. Omissions on contralesional side of the face indicate personal visuospatial neglect.	Used for bedside-screening rather than for clinical diagnosis.
Eyetracking oculography	Requires the patient to focus at the midpoint of a given object, image, or task. An orientation bias towards the ipsilesional side indicates visual visuospatial neglect.	Pure assessment of visual visuospatial neglect as no manual exploration is necessary. Does not assess other aspects of visuospatial neglect.
Test for attentional performance (TAP): subtest ‘Neglect’	Pressing a button when a peripheral flicker stimulus appears on a screen together with distractor stimuli. Omissions on the contralesional side indicate visuospatial neglect.	If a patient suffers from HH, visuospatial neglect cannot be diagnosed with this test.
Test for attentional performance (TAP): subtest ‘Covert Attention’	Reacting to stimuli presented on either side of the screen preceded by a valid or invalid cue stimulus. Visuospatial neglect is indicated by reaction times in invalid trials towards the contralesional hemi-field that are even more prolonged than in healthy subjects.	Used to differentiate visuospatial neglect from visual field deficits.
Virtual reality	Exploration of a virtual environment conveyed through a head-mounted display. The system tracks eye and head movements.	This allows the safe assessment of ADL in a virtual environment. More information on validity, reliability, or sensitivity is required.
Clinical observation and information from relatives	Behavioural observation (e.g., during self-care, dressing, eating, or in therapeutic settings). This should also include information obtained from care-givers.	Adds important diagnostic value.

Adapted from Plummer, Morris, and Dunai (2003) and Ting et al. (2011). *Abbreviations*: ADL: Activities of daily living; HH: homonymous hemianopia.

## Therapy

Endogenous plasticity after stroke leads to sub-acute recovery in approximately two thirds of patients only (Karnath, Rennig, Johannsen, & Rorden, 2011), leaving a substantial proportion of patients with chronic visuospatial neglect, that is associated with reduced independence and lasting disability (Jehkonen, Laihosalo, & Kettunen, 2006). Rehabilitation techniques aim to retrain functions (restitution), utilize remaining intact brain structures (compensation), or adapt to impairments by using external devices or modifications (substitution: see Table 3) and can be categorized into top-down or bottom-up approaches (Parton, Malhotra, & Husain, 2004).

**Table 3:** Treatment of visuospatial neglect

Types	Interventions	Brief descriptions
Compensation	Visual scanning	Improve scanning patterns by learning systematic search strategies using paper-pencil and large screen projections.
	Optokinetic stimulation	Induce left pursuit movement with the aid of leftward moving background targets and hereby shifting attention to the contralesional hemi-space. Can be combined with other interventions.
	Limb activation	Improve attention toward the neglected hemifield by moving the contralesional limb in the neglected hemispace.
	Cueing	Activate attention towards neglected side with “cueing” stimuli (visual, auditory or tactile).
	Spatial perception	Feedback-related training of visuospatial deficits, reduction of uncertainty in space perception.
	Neck muscle vibration	Re-center the spatial egocentric frame of reference by modifying the afferent neck proprioceptive inputs relative to the position of the head to the trunk.
	TENS	Re-center the spatial egocentric frame of reference by transcutaneous electroneural stimulation of the left neck muscles with a low-voltage current.
	Trunk rotation	Re-center the spatial egocentric frame of reference by modifying the afferent information relative to the position of the head to the trunk.
	Caloric (vestibular) stimulation	Induce nystagmus towards the affected side with cold contralesional or warm ipsilesional caloric stimulation of the ear.
	Eye patches	Increase leftward saccades by occluding the unaffected right hemifield.
	Fresnel prisms	Shift the affected visual hemifield towards the unaffected ipsilesional egocentric frame of reference.
	Sustained attention	Improve spatial attention by activating the arousal system (using alerting stimuli).
Substitution	Prism adaptation	Re-center the spatial egocentric frame of reference by using prism adaptation and successive visuo-motor actions (exploiting the after-effect of leftward shift towards the neglected hemifield).
	Diminished background pattern and foreground clutter	Minimize the required visual attention during visual scene navigation by reducing background and foreground environmental distractors.
Restitution	Pharmacological treatment	Improve attention by using dopaminergic, noradrenergic or pro-cholinergic drugs.
	Mental imagery	Improve representational (imagery) neglect by using visual or movement imagery.
	VR space remapping	Remap the egocentric reference frame towards the affected hemifield by using VR systems.
Compensation and restitution	Feedback training	Increase patients’ self awareness (restitution) with feedback sessions (video, mirror, verbal, visuo-motor) following certain tasks, pointing out their behaviour, and teaching them “compensatory” strategies.
	TMS and tDCS	Disrupt the integrated neuronal acitvity of the intact side, dampen the attention to the ipsilesional side and thereby restore orientation balance between both hemispheres (compensation), or alternatively, induce the process of neuroplasticity (restitution).
Unknown	Music therapy	Stimulation via sensory and emotional pathways.

Adapted from Kerkhoff (2001), Ting et al. (2011)

*VR* virtual reality, *rTMS* repetitive transcranial magnetic stimulation, *tDCS* transcranial direct current stimulation, *TENS* Transcutaneous electroneural stimulation

A Cochrane review (Bowen et al. 2013) investigating the effects of various cognitive rehabilitation interventions in 23 randomized controlled trials concluded that there is insufficient evidence to adequately evaluate the effect of cognitive rehabilitation on functional ability or even standardized visuospatial neglect assesment. However, this review omitted pharmacological treatment, neck muscle vibration, vestibular stimulation, virtual reality and noninvasive brain stimulation. In a recent review on rehabilitation of visuospatial neglect, Azouvi et al. (2017) concluded, that there still is only a low level of evidence for various rehabilitation methods and emphasized the need for larger validation trials using innovative and promising rehabilitation techniques such as noninvasive brain stimulation.

Pharmacological treatment has been investigated in animals and humans, but the literature is sparse. In animals, visuospatial neglect can be induced by unilateral interruption of the dopaminergic system and subsequent administration of dopamine agonists can reduce visuospatial neglect (Corwin et al., 1986). In humans, an improvement of visuospatial neglect symptoms was reported with dopamine agonists (Fleet, Valenstein, Watson, & Heilman, 1987; Mukand et al., 2001). Other pharmacological approaches include noradrenergic agents (Malhotra, Parton, Greenwood, & Husain, 2006) and pro-cholinergic drugs (Thiel, Zilles, & Fink, 2005). To date, no valid conclusions can be drawn and pharmacological treatment is usually not part of the rehabilitative strategy (van der Kemp, Dorresteijn, Ten Brink, Nijboer, & Visser-Meily, 2017).

A majority of therapeutic strategies leads to transitional improvements of symptoms including transfer to non-trained activities and ADL. However, therapeutic strategies may not target the underlying dysfunctions of visuospatial neglect appropriately as those are not yest sufficiently understood. Further improvement of diagnostic as well as therapeutic tools may have a great impact on rehabilitation outcome and costs. The improvement of therapeutic means furthermore critically depends on the investigation of the underlying neuropsychological, physiological, and anatomical factors.

## The Role of Noninvasive Stimulation in the Treatment of Visuospatial Neglect

**Fig. 2:**
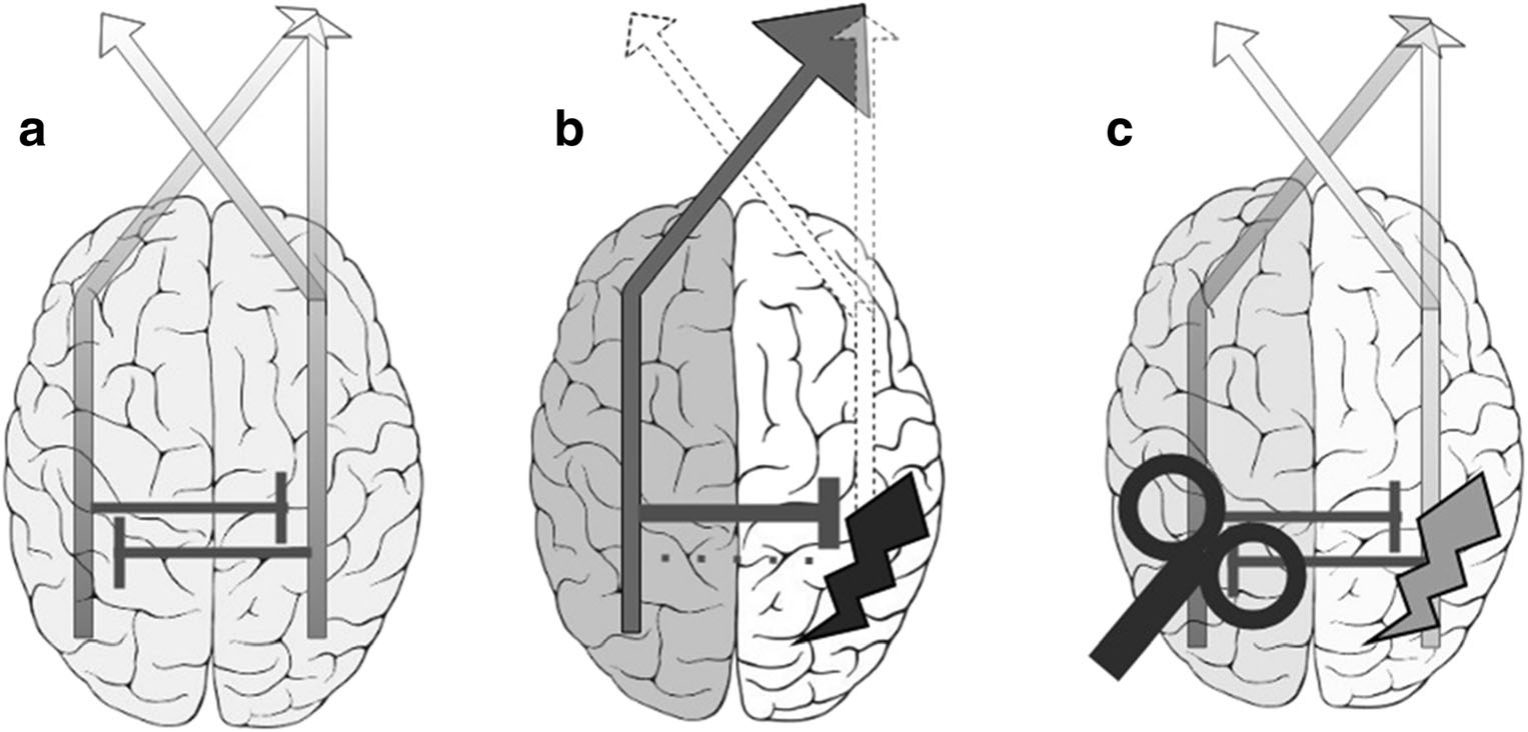
Interhemispheric rivalry model and noninvasive brain stimulation: **a** Balanced allocation of attention: In healthy subjects, allocation of attention towards both hemi-spaces is balanced by mutual transcallosal inhibition. Note, that the right hemisphere regulates attention towards both the left and right hemispace, which might offer an explanation for the higher prevalence of visuospatial neglect after right-hemispheric lesions. **b** Dysbalance following unilateral lesion: Righthemispheric damage leads to hypoactivity of the lesioned right and hyperactivity of the intact left hemisphere. **c** Attenuated dysbalance induced by noninvasive brain stimulation: Brain Stimulation (e.g., low-frequency TMS over the left hyperactive hemisphere) can attenuate interhemispheric dysbalance and ameliorate visuospatial neglect

Noninvasive brain stimulation is a means to explore and modulate human brain functions. The most commonly used forms of noninvasive brain stimulation are transcranial magnetic stimulation (TMS) and transcranial direct current stimulation (tDCS), which are safe and can be applied in a wide range of patients with neurological and psychiatric disorders (Bikson et al., 2016; Rossi et al., 2009). In stroke patients, noninvasive brain stimulation is used to modulate motor (Hummel et al., 2005) and non-motor symptoms (Elsner, Kugler, Pohl, & Mehrholz, 2016), including visuospatial neglect (Müri et al., 2013). Excitatory noninvasive brain stimulation protocols include high-frequency TMS, intermittent theta-burst-stimulation (iTBS), and anodal tDCS, while inhibiting protocols like low-frequency TMS, continuous TBS (cTBS) and cathodal tDCS seem to decrease neuronal excitability under the stimulation site. The use of noninvasive brain stimulation for visuospatial neglect rehabilitation is based on the above-described “Interhemispheric Rivalry Model” (Kinsbourne, 1970, 1987, 1993). Excitatory and inhibiting protocols are employed to restore interhemispheric balance (see Fig. 2). However, future stimulation protocols might administer individually designed, multi-site, network-based protocols. In the following sections, we present and discuss evidence that presents noninvasive brain stimulation as a diagnostic tool to explore the pathophysiology of visuospatial neglect, as well as promising treatment results.

### Studies with Animals

Animal research shows that unilateral lesioning or cooling of critical parietal regions could induce visuospatial neglect-like symptoms in cats (Lomber, Payne, Hilgetag, & Rushmore, 2002; Rushmore, Valero-Cabre, Lomber, Hilgetag, & Payne, 2006; Sprague, 1966). Resulting interhemispheric imbalance could subsequently be lessenend by reducing contralesional hyperexcitability, thus providing support for the interhemispheric rivalry model. More recently, cathodal tDCS (Rushmore, DeSimone, & Valero-Cabré, 2013) and repetitive TMS (rTMS; Afifi, Jarrett Rushmore, & Valero-Cabré, 2013) were used to ameliorate induced visuospatial neglect symptoms in cats, resulting in lasting effects of recovery.

### Studies with Healthy Subjects

Bjoertomt, Cowey, and Walsh (2002) showed that low-frequency rTMS over the right PPC or the right ventral occipital lobe selectively induced a rightward shift in the perceived midpoint for horizontal lines in healthy subjects. This is in line with the interhemispheric rivalry model, as low-frequency TMS is believed to induce inhibition and high-frequency TMS to induce enhanced cortical responses, or facilitation. Similarly, single pulse TMS (believed to disturb cortical processing) over the right PPC induced a significant rightward bias in a line-length judgment task (Fierro et al., 2006). In another study, unilateral single pulse TMS over the left and right PPC impaired detection of contralaterally presented visual stimuli (Dambeck et al., 2006). Interestingly, biparietal stimulation over both PPCs resulted in unimpaired stimulus detection, hereby providing further support for the interhemispheric rivalry model. Using cTBS over the right PPC, Nyffeler et al. (2008) induced sustained visuospatial neglect-like behavior in a visual exploration task lasting up to 30 min. In a subsequent cTBS-experiment, they were able to reverse visuospatial neglect-like behavior by stimulating over the left PPC (Dario Cazzoli, Wurtz, Müri, Hess, & Nyffeler, 2009).

tDCS studies provide additional support for the interhemispheric rivalry model. As anodal stimulation is thought to increase, and cathodal stimulation to decrease cortical excitability, polaritiy-specific visuospatial impairments have been hypothesized to arise after concomitant stimulation of both PPCs (e.g., simultaneous anodal left and cathodal right stimulation were used to mimic a right-sided lesion). Indeed, visuospatial neglect-like difficulties in symmetry judgement (Giglia et al., 2011), visuospatial localization (Wright & Krekelberg, 2013), and perceived position (Wright & Krekelberg, 2014) could be observed. Using anodal and cathodal stimulation over the left or right PPC in separate sessions, Sparing and colleagues could enhance or impair the detection of a target presented contralaterally (with respect to the tDCS site; Sparing et al., 2009).

More recent work combined low-frequency rTMS over the intraparietal sulcus with subsequent functional magnetic resonance imaging (fMRI). Plow et al. (2014) observed an activation-pattern of interhemispheric imbalance in accordance with the interhemispheric rivalry model. In a subsequent experiment, the same group revealed large scale intra- and interhemispheric changes in functional connectivity after low-frequency rTMS (Battelli, Grossman, & Plow, 2017), which not only provides further support for the interhemispheric rivalry model, but also for frontoparietal network-models outlined above. In another study to explore neural mechanisms of attentional control, Szczepanski and Kastner (2013) used online single-pulse TMS during a spatial attention task. Simultaneous single-pulse TMS to both PPCs resulted in no behavioral bias, while stimulation over either PPC resulted in ipsilateral attention biases. Importantly, Szczepanski and Kastner (2013) also found substantial interindividual differences in baseline spatial bias and in behavioral response to varying stimulation parameters – an important finding, which implies that future research should focus on the optimization of individually tailored protocols.

Studies in healthy subjects support the hypothesis of a rivalry of spatial attention between the two hemispheres and emphasize the role of the right parietal cortex in spatial processing. However, as these results have been obtained in young subjects, their translational value for the use of noninvasive brain stimulation in rehabilitation of visuospatial neglect is limited. Age affects the lateralization of task-related activity in healthy subjects (Cabeza, 2002) and further issues arise when stroke patients are stimulated, as lesions affect current distribution in the brain (Wagner et al., 2007). Furthermore, these studies predominantely stimulated the PPC, assuming it to be the most important area in spatial processing, though recent research suggests that subcortical structures and possible diaschisis effects play an important role within this network. Unfortunately, to date subcortical structures can only be targeted indirectly with noninvasive brain stimulation. Moreover, other parts of the network, such as inferior frontal regions or the frontal eye fields, may be important in later processing stages and their contribution should be investigated in future studies.

### Studies with Stroke Patients: Systematic Review

Noninvasive brain stimulation is not only applied as a research tool to improve the pathophysiological understanding of visuospatial neglect, but also as a treatment device. Based on the interhemispheric rivalry model, several stimulation approaches can be deduced: excitation of the lesioned hemisphere, inhibition of the non-lesioned – but hyperactive – hemisphere, or a combination of both. Due to safety concerns regarding the potentially increased risk of seizures, most TMS-studies focussed on inhibiting the non-lesioned hemisphere. The majority of studies used TMS-protocols, but in recent years a few studies using tDCS-protocols were published. To evaluate the efficiacy of noninvasive brain stimulation in post-stroke visuospatial neglect, we conducted a systematic review adhering to the Preferred Reporting Items for Systematic Reviews and Meta-Analyses (Moher et al., 2015).

Reflecting the multifaceted nature of visuospatial neglect, a wide range of outcome measures was employed. Further complicating comparability among studies, a variety of stimulation parameters (stimulation site, amount of sessions), study designs and inclusion criteria (e.g., time since stroke) were applied. To our knowledge, no studies to date explored novel electrical stimulation methods such as transcranial random noise stimulation (tRNS) or transcranial alternating current stimulation (tACS) in patients with visuospatial neglect. Furthermore, more recently developed deeper-reaching coils like H-coils or double-cone-coils have not been applied in patients with visuospatial neglect.

#### Search Strategy and Information Sources

We searched MEDLINE, EMBASE and CENTRAL (most recent search in April 2019, no historical limit applied). Medical-Subject-Headings (MeSH)-Terms and direct entries were used. We additionally screened reference lists of relevant articles. The search strategy was as follows: ((Brain stimulation OR Brain-stimulation OR tDCS OR Transcranial Direct Current Stimulation OR Transcranial-Direct-Current-Stimulation OR Cathodal Stimulation OR Anodal Stimulation OR Random Noise Stimulation OR Random-Noise-Stimulation OR RNS OR tRNS OR Alternating Current OR Alternating-Current OR tACS OR Transcranial Magnetic Stimulation OR Transcranial-Magnetic-Stimulation OR TMS OR Theta Burst Stimulation OR Theta-Burst-Stimulation OR TBS OR iTBS OR cTBS OR H-Coil OR Double-Cone-Coil)) AND (Visuospatial Neglect OR Visuospatial Neglect OR Hemispatial Neglect OR Hemi-spatial Neglect OR Unilateral neglect OR Uni-lateral Neglect OR Visual Neglect OR Hemispheric Neglect OR Hemineglect OR Hemi-Neglect OR Attentional Neglect OR Spatial Neglect OR Sensory Neglect)).

#### Eligibility Criteria

We included peer-reviewed cohort studies and case reports of stroke patients with visuospatial neglect written in English language, in which noninvasive brain stimulation was used as the primary intervention to treat visuospatial neglect. Due to the heterogenity of visuospatial neglect and diagnostic difficulties (see section 6; “Diagnosis”), no restrictions regarding diagnostic criteria or outcome measures were applied.

#### Results

**Fig. 3:**
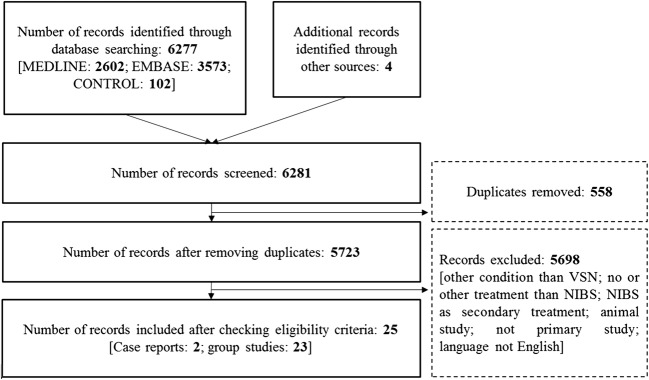
Flow-chart of the systematic review following PRISMA guidelines (Moher et al., 2015). A total of 6281studies were found using the described search string and through other sources. Twenty five studies fulfilled eligibility criteria and were included in the review

The database search resulted in 6277 hits. Four more publications were added afterwards. In total, 25 studies (12 randomized controlled trials, two case studies) published between September 1999 and April 2019 were included in the systematic review (Fig. 3 and Table 4).

**Table 4:** Clinical trials and case studies assessing effects of noninvasive brain stimulation on symptoms of visuospatial neglect

	Design	Total sample/ weeks post-stroke	Stimulation site	Intensity/ current	Number of pulses per session/Duration	Number of sessions	Tpye and timing of visuospatial neglect-assessment	Main results
*rTMS*								
Oliveri et al. (2001)	within-subject, cross-over: Stim vs sham	*n* = 7 / 15.7^a^ w (1–48)^b^	contralesional PPC	115% of motor threshold	10 pulses at 25 Hz	single session	LB; during stim	↓ Ipsilesional bias in LB
Brighina et al. (2003)	within-subject, uncontrolled	*n* = 3 / 14.6 w (12–20)	contralesional PPC	90% of motor threshold	900 pulses at 1 Hz	7 sessions over 14 d	LB; 15 d before, at beginning, at end and 15 d after stim period	↓ Ipsilesional bias in LB after stim and at FU
Shindo et al. (2006)	within-subject, uncontrolled	n = 2 / 24 w	contralesional PPC	95% of motor threshold	900 pulses at 0.9 Hz	6 sessions over 2 w	BIT; 2 w before, 1d before, 1 d after, 2 w after, 4 w after, 6 w after stim period.	↑ BIT-scores after stim and at each FU
Koch et al. (2008)	within -subject, uncontrolled	*n* = 12 / 4–24 w (no individual data provided)	contralesional PPC	90% of motor threshold	600 pulses at 1 Hz	single session	Naming of chimeric objects; before and after stim	↑ Correctly named objects after stim
Song et al. (2009) >RCT	between-subject; Stim+rehab vs rehab only	*n* = 14 (7 vs 7)^c^ / 4.7 w (2–9)	contralesional PPC	90% of motor threshold	15 min at 0.5 Hz (450 pulses)	28 sessions over 14 d	LB, CT; 2 w before, at the beginning, at the end, 2 w after stim period.	↓ Ipsilesional bias in LB, ↓ omissions in CT in experimental, but not control group after stim and at FU
Lim, Kang, and Paik (2010)	between-subject: stim+rehab vs rehab only (retrospectively selected)	n = 14 (7 vs 7) / 14.3 w (1–67)	contralesional PPC	90% of motor threshold	900 pulses at 1 Hz	10 sessions over 2 w	LB, Albert test; 1 d before and 1d after stim period	↑ Improvement in LB stim group
Kim, Chun, Kim, and Lee (2013) >RCT	between-subject: low-frequency vs high-frequency vs sham	*n* = 27 (9 vs 9 vs 9) / 2.1 w (no individual data provided)	low frequency over contralesional PPC, high-frequency over ipsilesional PPC	90% of motor threshold	-low frequency: 1200 pulses at 1 Hz -high-frequency: 1000 pulses at 10 Hz	10 sessions over 2 w	MFVPT, LB, CT, CBS; before and after stim period.	↑ Improvement in LB for high-fequency-stim compared to sham.
Cha and Kim (2015a) >RCT	between-subject:Stim+rehab vs sham+rehab	*n* = 30 (10 vs 10 vs 10)/ 16.3 w (no individual data provided)	contralesional PPC	90% of motor threshold	1200 pulses at 1 Hz	20 session over 4 w	LB, Albert test; before and after stim period.	↑ Improvement in LB in experimental group
Cha and Kim (2015b) >RCT	between-subject:stim+rehab vs sham+rehab	*n* = 20 (10 v 10)/ 4.6 w (no individual data provided)	contralesional PPC	90% of motor threshold	600 pulses at 1 Hz	20 session over 4 w	MFVPT, LB, Albert Test, Star CT; before and after stim period.	↑ Improvement in LB and MFVPT in experimental group
Yang et al. (2015) >RCT	between-subject: low-frequency + rehab vs high-frequency + rehab vs cTBS+rehab vs sham+rehab	*n* = 38 (9 vs 10 vs 9 vs 10) / 14.8 w (no individual data provided)	contralesional PPC	80% of motor threshold	-low frequency: 656 pulses at 1 Hz -high-frequency: 1000 pulses at 10 Hz -cTBS: 801 pulses (bursts of 3 pulses at 30 Hz)	28 sessions over 4 w	CT, LB; 2 w before, at the beginning, at the end, 1 m after stim period.	↑ CT-scores at FU for stim groups (cTBS>1 Hz > 10 Hz) compared to baseline ↓ Ipsilesional bias in LB at FU for cTBS and 1 Hz group compared to baseline
Yang, Fong, Wp Li-Tsang, and Zhou (2016) >RCT	between-subject: stim+rehab vs stim+ cueing+rehab vs sensory cueing+rehab vs rehab only	*n* = 60 (20 vs 20 vs 20)/ 5.5 w (no individual data provided)	contralesional PPC	90% of motor threshold	900 pulses at 1 Hz	10 sessions over 2 w	BIT, CBS; before, 2w after, 6w after stim period.	↑ Improvement in BIT-scores for stim+cueing group compared to no stim after stim and at FU ↑ Improvement in BIT-scores for stim only group compared to no stim at FU
*spTMS*								
Oliveri et al. (1999)	within-subject, frontal vs prefrontal vs parietal stim	n = 6 / 4.4 w (4–8)	contralesional frontal, prefrontal, parietal cortex	110% of motor threshold	single pulse	single session (45 trials)	Extinction test (sensitivity to cutaneous stim); followed by stim after 40 ms	↓ Extinction during frontal and prefrontal stim
*cTBS*								
Nyffeler, Cazzoli, Hess, and Muri (2009)	within-subject: stim vs sham vs no intervention	*n* = 11 / 28.4 w (2–144)	contralesional PPC	100% of motor threshold	1602 (2 trains) to 2404 (4 trains) pulses (bursts of 3 pulses at 30 Hz repeated at 10 Hz)	single session	Peripheral visual attention task; before, 1, 3, 8, 24, 32, 96 h after stim.	↑ Improvement in peripheral visual attention task for up to 8 h after 2 trains and up to 32 h after 4 trains in stim condition
Cazzoli et al. (2012)	between-subject: stim followed by sham vs sham followed by stim vs no intervention	*n* = 24 (8 vs 8 vs 8) / 3.8 w (no individual data provided)	contralesional PPC	100% of motor threshold	2404 pulses - 4 trains (bursts of 3 pulses at 30 Hz repeated at 6 Hz)	2 sessions over 2 d	CBS, CT, reading task, two part picture test, peripheral perception task; before, after stim and sham, 2 w after stim	↑ Improvement in CBS-scores and peripheral perception in both stim groups compared to no-stim after stim and at FU
Koch et al. (2012) >RCT	between-subject: stim vs sham	*n* = 18 (9 vs 9) / 6.2 w (4–15)	contralesional PPC	80% of motor threshold	1200 pulses (bursts of 3 pulses at 50 Hz, repeated at 5 Hz)	10 session over 2 w	BIT; before, after, 4 w after stimulation period	↑ BIT-scores for stim compared to sham after stim and at FU
Fu et al. (2015) >RCT	between-subject:stim+rehab vs sham+rehab	n = 20 (10 vs 10)/ 6.1 w (2–16)	contralesional PPC	80% of motor threshold	2400 pulses – 4 trains (bursts of 3 pulses at 30 Hz, repeated at 5 Hz)	14 sessions over 14 d	CT, LB; before, after, 4 w after stim period	↑ CT-scores for stim compared to sham after stim ↓ Ipsilesional bias in LB for stim compared to sham at FU
Fu et al. (2017) >RCT	between-subject: stim+visual scanning vs low-intensity stim+visual scanning	n = 12 (6 vs 6) / 5.6 w (no individual data provided)	contralesional PPC	80% of motor threshold vs 40% of motor threshold (control condition)	2400 pulses - 4 trains (bursts of 3 pulses at 30 Hz, repeated at 5 Hz)	10 sessions over 10 d	CT, LB; before and after stim period.	↓ Ipsilesional bias in LB, ↑ CT-scores for stim compared to sham after stim
*iTBS*								
Cao et al. (2016) >RCT	between-subject: stim+visual scanning vs low-intensity stim+visual scanning	*n* = 13 (7 vs 6)/ 4.8 w (no individual data provided)	contralesional DLPFC	80% of motor threshold vs 40% of motor threshold (control condition)	600 pulses – 20 short trains (bursts of 3 pulses at 50 Hz repeated at 5 Hz)	20 sessions over 10 d	LB, CT; before and after stim period	↑ Improvement in LB, CT in high-intensity- compared to low-intensity-group
*tDCS*								
Ko, Han, Park, Seo, and Kim (2008)	within -subject, crossover: Stim vs sham	*n* = 15 / 4–14 w (no individual data provided)	ipsilesional PPC (anodal)	2.0 mA	20 min	Single session	LB, CT; before and immediately after stim	↑ Improvement in LB, CT in stim, but not in sham condition
Sparing et al. (2009)	within-subject, crossover: left anodal vs left cathodal vs right anodal vs right sham stim	*n* = 10 / 11.6 w (no individual data provided)	ipsilesional PPC (anodal, sham), contralesional PPC (anodal, cathodal)	1.0 mA	10 min	Single session	TAP subtest ‘neglect’, LB; before, immediately after, 20 min after stim	↓ Ipsilesional bias after stim in left cathodal and right anodal condition
Sunwoo et al. (2013)	within-subject, crossover: dual (right anodal+left cathodal) vs right anodal vs sham	n = 10 / 111.2 w (4–784)	ipsilesional PPC (anodal), contralesional PPC (cathodal)	1.0 mA	20 min	Single session	LB, CT; before, immediately after stim	↓ Ipsilesional bias for stim groups compared to sham (dual>anodal>sham)
Brem, Unterburger, Speight, and Jäncke (2014)	within-subject, crossover: cognTr vs cognTr+sham vs cognTr+dual stim	n = 1 / 4 w	ipsilesional PPC (anodal), contralesional PPC (cathodal)	1.0 mA	20 min	Single session and 5 sessions over 5 d	TAP subtest ‘covert attention’, LB, CT, copying figures, ADL-questionnaire; before and after single stim sessions, 1 w, 3 m after stim period	↑ Improvement in covert attention task, qualitative improvement in LB und coyping after stim compared to sham. ↑ Improvement in ADL at 3 m-FU
Bang and Bong (2015) >RCT	between-subject: dual stim+feedback training vs feedback training	n = 12 (6 vs 6)/ 6.7 w (no individual data provided)	ipsilesional PPC (anodal), contralesional PPC (cathodal)	1.0 mA	20 min	15 sessions over 3 w	MFVPT, LB; before and after stim period	↓ Ipsilesional bias in LB, ↑ MFVPT-scores in experimental group compared to control group after stim
Smit et al. (2015)	within-subject: dual stim vs sham	*n* = 5 / 230.4 w (54–592)	ipsilesional PPC (anodal), contralesional PPC (cathodal)	2.0 mA	20 min	5 sessions over 5 d	LB, CT, drawing; before and after stim period	Null result
Yi et al. (2016) >RCT	between-subject: left cathodal vs right anodal vs sham	n = 30 (10 vs 10 vs 10)/ no information provided	ipsilesional PPC (anodal), contralesional PPC (cathodal)	2.0 mA	30 min	15 sessions over 3 w	MFVPT, LB, CT, CBS; before and 1 w after stim period	↑ Improvement in MFVPT, LB, CT for both stim conditions compared to sham. No difference between stim groups

^a^*= mean,*^*b*^ *= range,*^*c*^ = number of patients in respective groups, RCT = randomized controlled trial., PPC = posterior parietal cortex, DLPFC = dorsolateral prefrontal cortex, LB = line bisection, T = cancellation task, MFVPT = Motor-Free Visual Perception Test, CBS = Catherine Bergego Scale, BIT = Behavioral Inattention Test, ms = 1*10^*−3*^ s, min = minute(s), d = day(s), w = week(s), m = month(s), FU = follow up, mA = 1*10^*−3*^*Ampere, CognTr = Cognitive Training*

*ADL*: activities of daily living; *BIT*: Behavioral Inattention Test; *CBS*: Catherine Bergego Scale; cognTr: cognitive training; *cTBS*; continuous theta burst stimulation; d: day(s); *DLPFC*: dorsolateral prefrontal cortex; *FU*: follow up; *h*: hour(s); *Hz*: Hertz; *iTBS*: intermittent theta burst stimulation; *LB*: line bisection, *m*: month(s); *mA*: milliampere; *min*: minute(s); *MFVPT*: Motor-Free Visual Perception Test; ms: millisecond(s); n: number of participants; *NIBS*: noninvasive brain stimulation; *PPC*: posterior parietal cortex; *RCT*: randomized controlled trial; rehab: rehabilitation; *rTMS*: repetitive transcranial magnetic stimulation; *spTMS*: single-pulse transcranial magnetic stimulation; stim: stimulation; *TAP*: Test for Attentional Performance; *tDCS*: transcranial direct current stimulation; *TMS*: transcranial magnetic stimulation; vs: versus; *VSN*: visuospatial neglect; *w*: week(s).

##### TMS-Studies

TMS appears to be a promising way of ameliorating symptoms of visuospatial neglect as demonstrated in a considerable number of studies. Eighteen studies using TMS fit our inclusion criteria, which investigated a total of 331 patients with visuospatial neglect. The majority (*n* = 227) of these studies applied rTMS over the contralesional PPC. Five more recent studies applied cTBS over the contralesional PPC and only two studies applied stimulation over contralesional prefrontal areas, one using iTBS (Cao et al., 2016) and one using single pulse-TMS (Oliveri et al., 1999).

Oliveri et al. (1999) were the first to apply noninvasive brain stimulation in patients with visuospatial neglect. They found that application of single-pulse TMS over the contralesional left frontal region decreased the level of contralateral tactile extinction. Assessing visuospatial neglect more specifically, Oliveri et al. (2001) applied a single train of high-frequency TMS meant to disrupt activity (real and sham) over the contralesional parietal cortex of seven patients with visuospatial neglect during the execution of a line bisection task and found a reduced ipsilesional judgment bias in a line bisection task. Brighina et al. (2003) applied low-frequency rTMS over the contralesional parietal cortex during seven sessions in three patients and found significant immediate and long-term improvement in length judgement, clock drawing, and line bisection.

Similarly, Shindo et al. (2006) stimulated the unlesioned PPC with low-frequency rTMS over 6 sessions in two visuospatial neglect-patients and detected significant changes in BIT-scores.

Koch et al. (2008) examined 12 stroke patients with visuospatial neglect, 8 stroke patients without visuospatial neglect (all with right-hemispheric lesions), and 10 healthy controls. Using a twin-coil approach, they applied a conditioning TMS pulse over the left PPC followed by a test pulse over the left motor cortex (M1) and found that excitability of left PPC-M1 circuits was higher in visuospatial neglect patients than the other groups, suggesting dysbalanced hemispheric activity in visuospatial neglect. Subsequent low-frequency rTMS over left PPC normalized over-excitability of the left PPC-M1 circuit and improved visuospatial neglect symptoms. In another small-sample study, Lim et al. (2010) employed low-frequency rTMS over the contralesional parietal cortex over 10 sessions in 7 patients prior to behavioural therapy and found greater improvement in line bisection compared to 7 patients that served as a control group (behavioural therapy only).

In recent years, a considerable number of randomized controlled trials evaluated the efficacy of rTMS in visuospatial neglect in multi-session-designs (Cha & Kim, 2015b, 2016; B. R. Kim et al., 2013; Song et al., 2009; W. Yang et al., 2015; Y. H. Yang et al., 2016). Most of these studies stimulated the contralesional (left) PPC. Overall, improvements in visuospatial neglect-specific outcome measures such as line bisection were found, lasting up to 6 weeks. However, only two studies included measures of ecological validity (B. R. Kim et al., 2013; Y. H. Yang et al., 2016), of which only one reported an immediate significant improvement in ADL (B. R. Kim et al., 2013).

Nyffeler et al. (2009) were the first to test the effects of cTBS over the contralesional parietal cortex. The results showed that two cTBS trains had a significant impact on the perception of visual targets for up to 8 h, whereas four cTBS trains increased improvements lasting up to 32 h. In a subsequent study, the same group showed substantive improvements in ADL after application of 8 trains of cTBS over 2 days, which persisted for 3 weeks after the end of treatment (Cazzoli et al., 2012). Adding further promising evidence for the use of cTBS in visuospatial neglect, Koch et al. (2012) demonstrated that a 2-week course of cTBS over the contralesional left PPC was effective in reducing visuospatial neglect symptoms. Fu et al. (2015) employed an even more intensive protocol consisting of 14 consecutive days of 4 trains of cTBS over the contralesional left PPC and observed improvements in paper-pencil-tests for visuospatial neglect in comparison to sham-stimulation. Interestingly, 4 weeks post-treatment the effects had increased even further, suggesting ongoing neurophysiological effects after the end of stimulation. In a later study, Fu et al. (2017) randomized 12 patients to receive cTBS with an intensity of 80% of motor threshold or – as a control group – with an intensity of 40% of motor threshold and found further evidence for the efficiacy of cTBS over the unlesioned (left) PPC, as the treatment group showed larger improvements in behavioural visuospatial neglect-tests compared to the control group.

Cao et al. (2016) applied iTBS for 10 days over the left DLPFC and observed larger improvements in line bisection and star cancellation when stimulation was applied at 80% of the resting motor threshold compared to when it was applied at 40% of the resting motor threshold. Notably, this study applied iTBS (an excitatory protocol) over the unlesioned hemisphere and at first sight does not seem to be in accordance with the interhemispheric rivalry model. However, stimulation was carried out over the left DLPFC (and not the PPC), a key brain region involved in top-down attentional control (Silton et al., 2010). Excitatory stimulation over this area might therefore boost compensatory functions in visuospatial neglect, independently of interhemispheric rivalry.

In sum, the majority of TMS protocols targeted the contralesionsal parietal cortex with the goal of reducing hyperactivity and found associated improvements in visuospatial neglect symptoms. Of specific interest are the more recently investigated cTBS protocols, which might exert a stronger effect than low-frequency rTMS (W. Yang et al., 2015). This might be particularly interesting in the clinical context as significant therapeutic effects could be achieved in a shorter time period.

##### Transcranial Direct Current Stimulation-Studies

To date, seven studies investigated the impact of tDCS on visuospatial neglect (Table 3). Sparing et al. (2009) tested 10 patients with right hemispheric lesions with computerized neglect tasks before and after anodal (over the lesioned hemisphere) and cathodal (over the unlesioned hemisphere) tDCS. Both anodal and cathodal tDCS caused a significant reduction in rightward bias in line bisection. Ko et al. (2008) stimulated 15 patients with anodal tDCS above the right (lesioned) PPC and found improvements in both line bisection and shape cancellation. In another study, Sunwoo et al. (2013) implemented an interhemispheric dual stimulation approach by combining anodal tDCS over the lesioned with cathodal tDCS over the intact hemisphere. Results showed a reduction of bias in line bisection and star cancellation with a significantly larger effect for dual stimulation compared to single stimulation paradigms.

Going one step further, Brem et al. (2014) were the first to combine biparietal tDCS with cognitive rehabilitation. In their sham-controlled single-case-study, they found a significant improvement in various outcome measures after combined tDCS and cognitive rehabilitation, while cognitive rehabilitation on its own and repeated stimulation sessions over 5 days did not result in significant changes. Following this multi-dimensional approach, Bang and Bong (2015) used biparietal tDCS combined with feedback training in a group of 6 patients and detected larger improvements in line bisection and ADL compared to a group of patients that received feedback training only. Contrary to that, Smit et al. (2015) found no treatment-related effects of biparietal tDCS over the PPCs in a group of five patients. However, in this feasibility study only chronic post-stroke patients (in one case, 12 years and 4 months) were included. Noninvasive brain stimulation studies usually recruit patients in the sub-acute stage (less than 6 months post stroke), as cortical reorganization and neurological recovery mainly takes place in the first few months after stroke (Kwakkel, Kollen, & Lindeman, 2004). Recently, Yi et al. (2016) randomized 30 patients into an anodal, a cathodal, and a sham group and applied 15 sessions over a course of 3 weeks. They reported larger improvements for line bisection, star cancellation, and a motor-free visual perception test in the stimulation groups compared to sham stimulation, but not for several measures of ADL.

To conclude, these findings confirm that parietal tDCS can lead to an improvement of visuospatial neglect symptoms. Transcranial direct current stimulation protocols are more flexible in terms of stimulation sites than TMS protocols and these studies therefore more often chose bilateral setups than TMS studies.

#### Discussion

When these results are considered together, the therapeutic value of noninvasive brain stimulation in visuospatial neglect seems very promising. Except for one study that investigated exclusively chronic stroke patients (Smit et al., 2015), all reviewed studies reported improvements in at least one outcome measure, and none reported deterioration. However, the overall low number of subjects included (*n* = 414 subjects: *N* = 331 in TMS-studies, *n* = 83 in tDCS-studies), as well as symptom heterogenitiy, pose a general problem in studies investigating visuospatial neglect. Additionally, the conducted studies are heterogeneous with regard to study designs, inclusion criteria, time since stroke, and outcome measures, hampering comparability and making conclusions difficult.

To date, only two studies applied noninvasive brain stimulation over the frontal cortex (Cao et al., 2016; Oliveri et al., 1999), both with positive results. However, no study examined frontal versus parietal stimulation sites or the combined stimulation over both sites. One study compared the effectiveness of rTMS and cTBS (W. Yang et al., 2015), with cTBS showing larger improvements in paper-pencil-measures. Despite its shorter stimulation duration, cTBS appears to have a stronger impact than rTMS and therefore may be particularly appealing for clinical use. A higher number of trains lead to a non-linear increase of life-time during which functional effects were still significant (Cazzoli et al., 2012). To our knowledge, no comparisons between TMS and tDCS have been undertaken yet. Only one study evaluated anodal versus cathodal tDCS (Yi et al., 2016), no differences between stimulation groups were observed. One study compared dual tDCS to anodal tDCS (Sunwoo et al., 2013; dual tDCS showed a stronger effect on line bisection performance). Furthermore, recently emerging electrical stimulation methods such as tACS or tRNS have not been applied in visuospatial neglect yet. Both techniques are believed to interact with cortical oscillations and synchronization (Antal & Paulus, 2013), which are known to be altered in visuospatial neglect (Rastelli et al., 2013). These methods therefore might open new avenues of using noninvasive brain stimulation in visuospatial neglect. Also, the necessary number of sessions required to achieve long-lasting effects needs to be adressed, as to our knowledge no study varied this parameter. The studies that applied repeated stimulation sessions showed mostly promising and long-term effects, up to 6 weeks (Brem et al., 2014; Brighina et al., 2003; Cao et al., 2016; Cha & Kim, 2015b, 2016; W. Fu et al., 2015; B. R. Kim et al., 2013; Y. K. Kim, Jung, & Shin, 2015; Koch et al., 2012; Shindo et al., 2006; Song et al., 2009; W. Yang et al., 2015; Y. H. Yang et al., 2016).

Although elaborate models of visuospatial neglect pathophysiology exist, there are still considerable uncertainties regarding their overall validity in this highly heterogenous population of patients. As visuospatial neglect is a multi-faceted, diverse condition, the need for individualized treatment approaches may be especially high for those patients. With regard to treatment with noninvasive brain stimulation, however, variability in responsiveness is an emerging issue in the field of neuromodulation in general (López-Alonso, Cheeran, Río-Rodríguez, & Fernández-Del-Olmo, 2014) and stroke research in particular (Wiethoff, Hamada, & Rothwell, 2014). Recent findings indicate notable interindividual variability in spatial attention networks and related responsiveness to noninvasive brain stimulation. Hence, not all patients with visuospatial neglect can be expected to profit equally from one and the same noninvasive brain stimulation protocol. Further advancement in the understanding of the interindividual pathophysiology of visuospatial neglect along with the diagnostic power of novel imaging-methods could be exploited to promote individualized treatment regimens in visuospatial neglect. As MRI-methods are both cost and time intense, EEG, which has recently been described as a helpful tool to study the integrity of network functionality in visuospatial neglect (Fellrath et al., 2016; Yordanova et al., 2017), could prove a useful method in investigating individual network disruptions in patients with visuospatial neglect, which could then serve as a basis for stimulation setups that target entire networks.

In view of the high diversity and combination of symptoms in patients with visuospatial neglect, many open questions merit further investigation and must be addressed in future work. These studies should assess a broad range of visuospatial neglect symptoms, assess long term effects, compare the efficacy of different noninvasive brain stimulation methods and combinatory approaches (i.e., noninvasive brain stimulation in combination with cognitive/occupational/physical rehabilitation), compare different stimulation protocols (patterns and site of stimulation, duration, number of sessions), explore possible benefits of MRI-guided targeting, and further elucidate underlying processes of visuospatial neglect, for example by using single-subject designs.

## Conclusions

Visuospatial neglect is a frequent and disabling impairment after stroke. In light of its multifaceted nature with heterogenous manifestations, diagnosis and treatment pose challenges in clinical routine.

Normal performance in bedside testing or a single paper-pencil test does not rule out visuospatial neglect. If visuospatial neglect is suspected, it is crucial that different tests covering a wide range of symptoms are administered. Neuropsychological diagnosis should therefore be based on patient observation and comprehensive testing. Often disregarded, concomitant cognitive impairment in non-spatial attention and executive functioning must be addressed carefully in neuropsychological assessment and therapy.

A wide range of therapeutic approaches has been applied in visuospatial neglect, including cognitive, behavioural, and pharmacological therapy. However, there is no consensus as to which rehabilitation methods and combinations are most effective. In recent years, technological advances have led to a better understanding of underlying neurophysiological, and anatomical factors of visuospatial neglect. The role of large-scale networks in visuospatial attention and their disruption in visuospatial neglect is now widely recognized and, in the course of this, noninvasive brain stimulation has emerged as a promising approach to study visuospatial neglect pathophysiology and advance treatment. Noninvasive brain stimulation as a rehabilitation tool is based on the interhemispheric rivalry model, in which excitatory and inhibiting protocols are employed to reestablish interhemispheric balance. As interindividual variability in treatment response is an important issue in visuospatial neglect, future stimulation protocols should be based on individually designed, network-based protocols. For example, each patient should be stimulated over different stimulation targets in order to identify the protocol that is most likely to (a) lead to behavioural changes and (b) modify brain activity and connectivity toward “normalization”.

In this review we have discussed the broad and complex nature of visuospatial neglect, presented currently used diagnostic and therapeutic options with a special emphasis on noninvasive brain stimulation, and offered recommendations for future studies. We hope that this review therefore contributes valuable information to advance the investigation of visuospatial neglect.
